# 5G NB‐IoT System Integrated with High‐Performance Fiber Sensor Inspired by Cirrus and Spider Structures

**DOI:** 10.1002/advs.202309894

**Published:** 2024-03-09

**Authors:** Lijun Lu, Guosheng Hu, Jingquan Liu, Bin Yang

**Affiliations:** ^1^ Key Laboratory of Materials Physics of Ministry of Education School of Physics and Microelectronics Zhengzhou University Zhengzhou 450001 China; ^2^ National Key Laboratory of Science and Technology on Micro/Nano Fabrication Shanghai Jiao Tong University Shanghai 200240 China; ^3^ Department of Micro/Nano Electronics School of Electronic Information and Electrical Engineering Shanghai Jiao Tong University Shanghai 200240 China

**Keywords:** 5G NB‐IoT, bioinspired fiber sensor, human‐machine interaction, information transmission, polymer

## Abstract

Real‐time telemedicine detection can solve the problem of the shortage of public medical resources caused by the coming aging society. However, the development of such an integrated monitoring system is hampered by the need for high‐performance sensors and the strict‐requirement of long‐distance signal transmission and reproduction. Here, a bionic crack‐spring fiber sensor (CSFS) inspired by spider leg and cirrus whiskers for stretchable and weavable electronics is reported. Trans‐scale conductive percolation networks of multilayer graphene around the surface of outer spring‐like Polyethylene terephthalate (PET) fibers and printing Ag enable a high sensitivity of 28475.6 and broad sensing range over 250%. The electromechanical changes in different stretching stages are simulated by Comsol to explain the response mechanism. The CSFS is incorporated into the fabric and realized the human‐machine interactions (HMIs) for robot control. Furthermore, the 5G Narrowband Internet of Things (NB‐IoT) system is developed for human healthcare data collection, transmission, and reproduction together with the integration of the CSFS, illustrating the huge potential of the approach in human–machine communication interfaces and intelligent telemedicine rehabilitation and diagnosis monitoring.

## Graphical abstract

Trans‐scale conductive percolation networks are constructed on the high‐performance bionic crack‐spring fiber sensor (CSFS) with high sensitivity and broad sensing range for human–machine communication interfaces and long‐distance information transmission with the assistance of 5G NB‐IoT system.

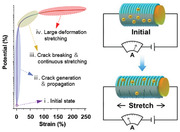

## 1 Introduction

In the era of artificial intelligence, flexible electronics have the potential to revolutionize various applications such as wearable e‐skins,^[^ ^1^, ^2^ ^]^ healthcare,^[^ ^3^, ^4^ ^]^ human–machine interface (HMI),^[^ ^5^, ^6^, ^7^ ^]^ and the Internet of Things (IoT).^[^ ^8^, ^9^ ^]^ According to IDTechEx forecasts, the flexible electronics industry is poised for sustained growth, estimated to be worth USD 301 billion in 2028. In particular, flexible wearable medical electronics can achieve long‐term seamless integration with human tissues and accurately measure clinical indicators, such as body temperature, respiration, blood pressure, and electrical activity, providing real‐time basic health data for big data‐driven medical treatments.^[^ ^10^, ^11^, ^12^ ^]^

The United Nations reported that the global population of older adults is growing by 2% per year, much faster than the total population. With an aging society on the horizon, public medical management capacity, including various medical resources, is becoming increasingly insufficient. The situation is exacerbated during infectious disease epidemics, when social distancing must be strictly followed in public places, especially hospitals. Realizing remote diagnosis and treatment of illness or injury at home will be of great benefit under such circumstances. Therefore, it is imperative to develop an integrated telemedicine system. Narrowband Internet of Things (NB‐IoT), which has low power consumption, wide coverage, and extensive connectivity, is increasingly recognized as a standard technology for 5G massive machine‐type communications.^[^ ^13^, ^14^ ^]^ With the gradual withdrawal of 2G and 3G networks, the NB‐IoT will provide massive connection services for low‐speed terminals. The combination of wearable sensors, 5G NB‐IoT, and artificial intelligence can achieve cloud collaboration and effectively solve problems involving data acquisition and transmission to support smart medical networking.

Currently, most stretchable electronics are fabricated using nonbreathable 2D elastic films.^[^ ^15^, ^16^ ^]^ However, healthy skin requires a microenvironment sufficiently permeable to air and moisture. Even at rest, the human body loses water at an evaporation rate of ≈600 g m^−2^ per day through insensible perspiration, which is essential for regulating body temperature.^[^ ^17^, ^18^ ^]^ Covering the skin with materials with insufficient moisture permeability can lead to discomfort. More concerningly, the long‐term use of electronic devices, especially those directly attached to the skin, can cause serious health problems, such as allergies and inflammation. In contrast, 1D electronic devices and systems, featuring a fiber‐shaped structure, are ideal for advanced wearable electronic devices owing to their unique characteristics such as flexibility, stretchability, and breathability.^[^ ^19^ ^]^ Furthermore, 1D stretchable electronics can be directly integrated into clothing, which is promising for future wearable devices.^[^ ^20^ ^]^

This promise has driven considerable research efforts toward improving the performance of flexible strain sensors through the development of new materials (such as graphene,^[^ ^21^, ^22^ ^]^ MXene,^[^ ^23^, ^24^ ^]^ metallic nanoparticles, and nanowires^[^ ^25^, ^26^ ^]^) or the design of special geometrical structures (such as gaps and island structures^[^ ^27^ ^]^ and percolation networks^[^ ^28^ ^]^). Nevertheless, developing sensors that combine the superiority of a large operational sensing range and high sensitivity remains a significant challenge in the pursuit of next‐generation wearable smart electronics.^[^ ^29^ ^]^ Fortunately, nature offers us instructive inspiration: microcracks on their legs providers spiders with super‐sensitivity,^[^ ^30^ ^]^ while cirrus whiskers with spontaneously generated spring‐like spiral structures allow some plants to climb and extend their reach. Therefore, by combining these two bionic structures, a sensor with a large detection range and high sensitivity can be realized simultaneously.

In this paper, we report a bionic crack‐spring fiber sensor (CSFS), inspired by the microcracks on a spider's leg and the cirrus whiskers in plants. This sensor enables the fabrication of stretchable and weavable electronics with high sensitivity and a large sensing range. The spring‐like core–sheath fiber, combined with polyethylene terephthalate (PET) fibers tightly wound around a highly elastic core rubber fiber, serves as a stretchable substrate. Multi‐layer graphene (Mgra) is ultrasonically deposited onto the surface of outer PET spring fibers, followed by the printing of ultrahigh‐conductivity Ag paste on one‐half of the Mgra/fiber along the axial direction. The Ag layer of the CSFS cracks under stress mismatch between itself and the Mgra/fiber base layer, generating a high sensitivity of 28475.6 and a large sensing range of over 250%. Typically, CSFS can be woven into fabric to realize an HMI for robot control. Moreover, as a proof of concept, remote medical diagnoses, including data collection, transmission, and reproduction, can be achieved by integrating the CSFS into an established 5G NB‐IoT system, demonstrating its great potential in human–machine communication interfaces and intelligent telemedicine.

## 2 Results and Discussion

### 2.1 Structure Characteristics and Fabrication of CSFS

**Figure 1:**
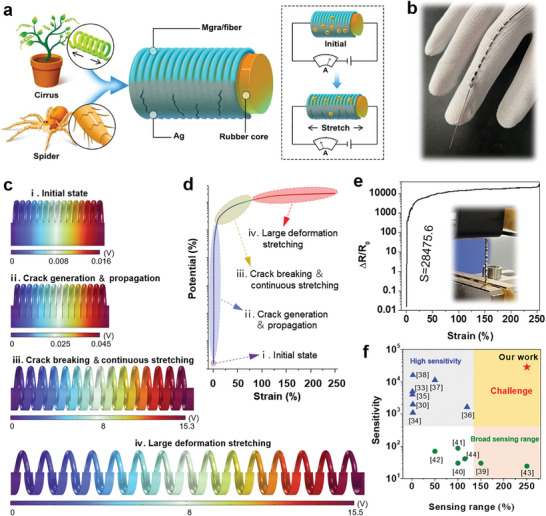
Design of the bionic crack‐spring coupled structure fiber sensor (CSFS). a) Schematic illustration of the CSFS, which consists of two main structures including the crack structure imitating spider leg and the spiral spring structure imitating vines, respectively. b), The CSFS sewn into the glove. c), Simulation of electric potential distribution with microstructure evolution during stretching process. d), The change of electric potential in different microstructure stages with different stretching degree. e), Typical relative resistance‐strain curve of the CSFS (inset, optical picture of the CSFS being tensile tested). f), Maximum sensing range and sensitivity of the CSFS compared with the previous sensors.

As illustrated in **Figure** **1**a, the bionic CSFS comprises two main structures: a crack structure imitating a spider's leg, and a spiral spring structure imitating vine. Specifically, the crack structure is key to achieving high sensitivity, whereas the spring‐like structure is beneficial for large‐range monitoring. With a diameter of only a few hundred microns, our fiber‐shaped CSFS can be easily sewn into wearable fabrics, such as gloves (Figure 1b). As a typical 2D nanomaterial, Mgra possesses excellent electrical conductivity, which makes it suitable for use in flexible electronic devices. Its clear multi‐layered structure can be observed in atomic force microscopy (AFM) and transmission electron microscopy (TEM) images, and its overall thickness is less than 10 nm (Figures S1 and S2, Supporting Information). Moreover, the relative ratio of *I*_G_/*I*_2D_ in the Raman spectrum also proves its multilayer structure, which is consistent with previous reports and morphological characterizations by AFM and TEM (Figure S3, Supporting Information).^[^ ^31^, ^32^ ^]^ The Mgra/fiber was continuously prepared via ultrasonic modification by wrapping the Mgra conductive networks around the outer spring‐like PET fibers. To verify the homogeneity of the ultrasonic treatment, the conductivities of the prepared Mgra/fibers of different lengths were measured. As seen in Figure S4 (Supporting Information), the fiber resistance exhibits a consistent linear increase with the increase in the length of the samples, which indicates uniform distribution and attachment of the Mgra to the fiber surface during this step. This provides a robust foundation for ensuring consistent sensor performance and enabling large‐scale production. For a wearable textile, it is essential to consider wash resistance. Therefore, we placed the prepared Mgra/fiber in a commercial washing machine to simulate a laundry scenario. It is evident from Figure S5 (Supporting Information) that, as the washing time progressed, the relative resistance of the samples changed only slightly, implying that the conductive networks were firmly constructed. Because the electromechanical functional layers were mainly concentrated in the outer spring Mgra/fiber and Ag layers, we used a simplified COMSOL simulation model, as shown in Figure 1c. The Ag conductive layer remained intact, and the potential difference between the two ends of the entire device was very small in the original state. With the stretching in stage ii, cracks were progressively created on the Ag layer, the potential difference increased rapidly, and the layer entered the resistance mutation region (as shown in stage ii of Figure 1d). Upon further stretching, the Ag conductive layer broke completely because of stress mismatch, at which point only the helical spring Mgra/fiber layer contributed to the conductive layer. Therefore, in stage iii, the potential difference increased by several orders of magnitude compared to the initial state and continued to increase linearly in small amplitudes with further stretching until reaching the maximum stretching state (as shown in stage iv of Figure 1c,d). Owing to the synergistic effect of the crack structure and the spiral spring‐like structure, the bionic CSFS demonstrated an ultrahigh sensitivity of 28475.6 in the small strain region (under 1%), while also exhibiting good detection capabilities for large strains over 250% (Figure 1e). However, it is difficult to improve these two factors for the same smart sensor. For example, the gauge factor (GF) of the Mgra/fiber device without the Ag crack layer was only ≈3 (Figure S6, Supporting Information). Therefore, combining these two excellent performances into one sensor enables the CSFS to fulfill a wider range of requirements for intelligent detection, thereby broadening its application prospects. These two key properties under stretching conditions are summarized in Figure 1f.^[^ ^30^, ^33^, ^34^, ^35^, ^36^, ^37^, ^38^, ^39^, ^40^, ^41^, ^42^, ^43^, ^44^ ^]^ Thus, we can conclude that, compared with other sensors of the same type, the CSFS better addresses the challenge of simultaneously enhancing the performance in these two parameters, which is a key step forward in advancing this technology.

**Figure 2:**
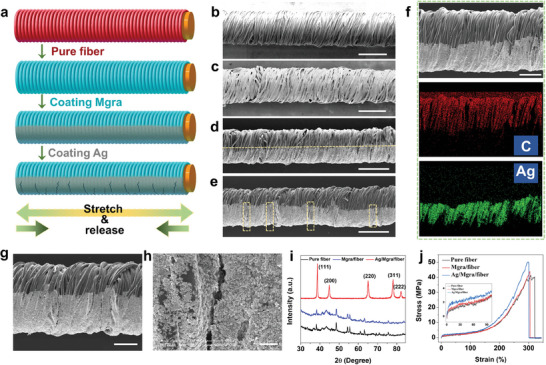
Fabrication and characterization of the CSFS. a), Schematic illustration of the fabrication process of the CSFS. b–d), SEM images of pure fiber, Mgra/fiber and Ag/Mgra/fiber, respectively. Scale bar: 500 µm. e), SEM image of the Ag/Mgra/fiber with cracks on Ag layer. f), SEM image and EDS mapping of the Ag/Mgra/fiber. Scale bar: 200 µm. g), Cracks of the Ag/Mgra/fiber. Scale bar: 200 µm. h), The enlarged view of (h). Scale bar: 20 µm. i), XRD spectrum of pure fiber, Mgra/fiber, and Ag/Mgra/fiber, respectively. j), Stress–strain curves of pure fiber, Mgra/fiber, and Ag/Mgra/fiber, respectively.

The fabrication process, as detailed in **Figure** **2**a, primarily included ultrasonic modification and printing. First, a flexible core–sheath structure fiber substrate with PET fibers tightly wound around a highly elastic rubber fiber was ultrasonically cleaned (Figure 2b). The adjacent PET fibers formed dense connections with each other, which separate and crack under small strains, while the core rubber fiber ensures remarkable stretchability. Conductive Mgra was decorated on the surfaces of the PET fibers (Figure 2c). As shown in Figure S7 (Supporting Information), the 2D Mgra was anchored evenly on the PET, forming a stable conductive path. Notably, the bending angle (0–180°) had no obvious effect on the conductivity of the Mgra/fiber, which greatly helped the subsequent preparation and processing (Figure S8, Supporting Information). Electrons can be transferred along the PET spiral or pass directly through two adjacent areas that are in contact. To achieve high sensitivity, the conductive Ag paste was printed on half of the Mgra/fiber surface along the axial direction, effectively reducing the resistance to the ohmic level as electrons can flow freely in the Ag conductive path (Figure 2d). However, because of the stress mismatch between the Ag metal layer and the Mgra/fiber base polymer layer, cracks were induced on the Ag layer of the CSFS, contributing to the ultrahigh sensitivity of the sensor (Figure 2e). SEM and elemental mapping images of the Ag/Mgra/fiber are shown in Figure 2f, in which the top and bottom hierarchical structures are clearly visible. Mgra appeared only in the upper half of the layer because the lower half was blocked by the Ag metal layer distributed in this area. Meanwhile, the magnified SEM images proved that a crack structure was formed on the Ag conductive layer when stretched, as mentioned above (Figure 2g,h).

To confirm the structure of the CSFS in each fabrication step, X‐ray diffraction (XRD) spectra were obtained. A few weak peaks appeared in the XRD spectra of the pure fiber and Mgra/fiber, which may be ascribed to trace impurities inherent in the substrate (Figure 2i). Five typical diffraction peaks representing the crystalline planes of (111), (200), (200), (220), (311), and (222) corresponding to Ag appeared in the Ag/Mgra/fiber, indicating the successful preparation of the CSFS. To investigate the tensile performance of the fibers, mechanical tests were conducted at different stages (Figure 2j). With the successive introduction of Mgra and Ag, the breaking strength of the pure fiber, Mgra/fiber, and Ag/Mgra/fiber increased to 41.32, 43.86, and 50.29 MPa, respectively. In contrast, the elongation at the break points exhibited a downward trend, decreasing to 320.74%, 305.03%, and 300.66%, respectively. This may be attributed to the fact that the addition of external substances increases the stress concentration and adversely affects elongation. In general, the deterioration of the mechanical properties is minimal after surface modification, with no fatal effect on the excellent elasticity of the CSFS.

### 2.2 Anti‐Interference Stability and Electromechanical Properties

**Figure 3:**
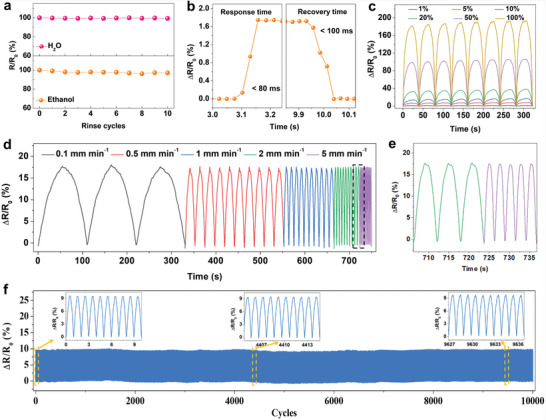
Anti‐interference stability and electro‐mechanical properties. a), Tolerance to water and alcohol of the CSFS. b), Response time and recovery time. c, Multicycle tests of relative resistance variation of the CSFS upon stretching to different strains. d), Relative resistance change of the CSFS under the tensile strain of 10% varies with the deformation rates. e), Partial enlarged version of (d). f), The durability test of the CSFS over 10 000 loading−unloading cycles under a strain of 5%.

Electrical stability against external interference is an important factor for evaluating sensor performance, and consistent characteristic response to strain is a crucial prerequisite for the accurate monitoring capability of a wearable resistive‐type sensor. Sensors are frequently exposed to various external factors; therefore, the effect of water and ethanol rinsing on the electrical conductivity of the CSFS was explored. The resistance of the CSFS remained nearly unchanged after 10 immersion cycles in either water or alcohol (**Figure** **3**a). Moreover, the resistance changed only slightly in different temperature and humidity environments, indicating that it could tolerate normal daily temperature and humidity variations (Figures S9 and S10, Supporting Information). This remarkable anti‐interference stability paves the way for high sensing performance and subsequent practical applications.

As shown in Figure 1f, the CSFS can combine two superior performances: high sensitivity (28475.6 at small strain) and a large sensing range (>250%). Owing to the stress mismatch, the conductive network structure is relatively less stable in the cracked area. Therefore, to investigate some basic electromechanical properties, the CSFS was pre‐stretched to extend it beyond the cracked area and into the spring strain region. The response time of the sensor has a significant influence on the application scenarios. For example, a severe sensor delay could lead to failure during relatively fast detection. The response curves in Figure 3b show that a short response time of 80 ms and a recovery time of 100 ms were achieved, fulfilling the requirements for normal wearable health and exercise monitoring of the human body.

To demonstrate whether the CSFS can satisfy various applications, the relative resistance responses to increasing strains of 1%, 5%, 10%, 20%, 50%, and 100% were measured cyclically after pre‐stretching to a 5% strain. As displayed in Figure 3c, a gradually increasing response with excellent repeatability was achieved for each strain. Besides, the relative resistance of the CSFS at a specific maximum strain of 10% remained almost unchanged when the deformation rate increased from 0.1 to 5 mm min^−1^ (Figure 3d,e), demonstrating its excellent adaptability to different external stimuli. Furthermore, the long‐term performance was studied in detail (Figure 3f). The cyclic responses of the CSFS remained remarkably well‐maintained for 10 000 cycles without any signs of fatigue (at a maximum strain of 5%), owing to the stability of the spring elastic structure during the stretching and releasing processes, indicating its excellent durability.

### 2.3 Human–Machine Interaction System for Gesture Recognition and Control

**Figure 4:**
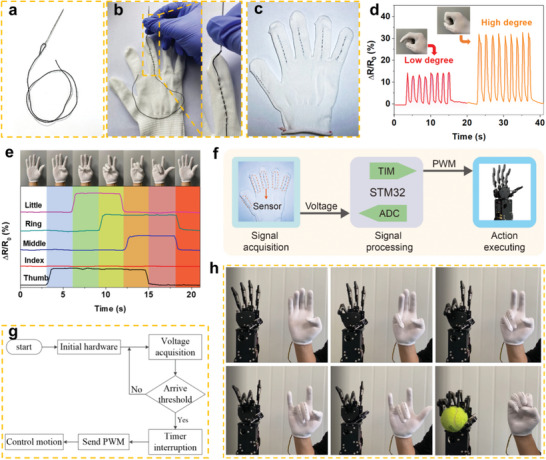
Human–machine interaction (HMI) system of gesture recognition and control. a), The CSFS being inserted into the eye of a needle. b), The sewing process. c), The as‐prepared smart glove. d), Real‐time monitoring of finger bending motion. e), The gesture recognition. f,g), Schematic diagram and flow chart of the human‐machine control system. h), The application of the CSFS to control the robotic hand.

Compared to a planar film device, the fiber‐shaped CSFS with a diameter of hundreds of microns was more suitable for wearable fabrics utilizing ancient weaving techniques. Benefitting from its flexibility and 1D shape, the CSFS could be easily inserted into the needle hole, greatly facilitating its sewing into garments (**Figure** **4**a). To illustrate this concept, the CSFS was inserted and fixed at each knuckle of a glove through simple sewing to monitor the complex gestures of human hands in real time (Figure 4b,c). The electrical signals corresponding to different gestures performed by the fingers wearing the smart glove were recorded instantaneously. For instance, owing to the excellent conformability between the CSFS and the body, as the finger bent rapidly at small or large deformation degrees, the response signals of the respective maximum amplitudes were accurately recorded, showcasing the fast response and recovery time of the sensor (Figure 4d). In addition, to evaluate the sensor's detection performance under sweating conditions, we simulated the conductive ionic condition in human sweat using a 0.5 wt.% NaCl solution, and the CSFS gloves were worn after washing hands without drying the surface of the residual NaCl solution. The electrical response signal remained largely unchanged under either large or small strains in the continuous finger‐bending test, as shown in Figure S11 (Supporting Information). This could be attributed to an insufficient amount of NaCl solution on the hand surface to fully saturate the CSFS and thus cause a significant change in its electrical response behavior. This finding provides valuable guidance for scenarios where monitoring under extreme sweating conditions is to be avoided. In addition to its effective dynamic response capability, the CSFS exhibited remarkable static response capability. As shown in Figure S12 (Supporting Information), when the finger was bent to a specific position and held, the feedback signal was also adjusted and maintained without any degradation, indicating the wide applicability of the CSFS in various complex situations. Remarkably, more complex gesture recognition can be realized by capturing the behavior of the array sensors installed on the five fingers, which also lays a solid foundation for the construction of HMI systems based on this wearable CSFS (Figure 4e).

Accurate perception of finger‐bending motion can extend the potential applications of the CSFS to control a robotic hand using HMI. As shown by the schematic diagram and flowchart shown in Figure 4f,g, the operation of gesture recognition and control was aided by the peripheral circuit modules comprising the acquisition, processing, and execution modules. The electric signal variations obtained by the motion of each finger were first transferred to quantitative analog signals. Subsequently, if the amplitude of the digital signals received through an analog‐to‐digital converter (ADC) was higher than a specific threshold, the executive module received specific commands. Therefore, the HMI system of gesture recognition and control was achieved based on this circuit and the designed flexible sensors. As shown in Figure 4h and Videos S1 and S2 (Supporting Information), accurate and real‐time gesture control of the robot hand was realized when the tester made different gestures, such as numbers four, three, and two. Notably, the robot hand can easily grab a tennis ball when the user makes a fist posture, demonstrating the tremendous potential of this system for application in more complex scenarios, such as substituting human intervention with indirect intelligent operations in high‐risk situations.

### 2.4 5G NB‐IoT System for Intelligent Telemedicine Rehabilitation and Diagnosis Monitoring

**Figure 5:**
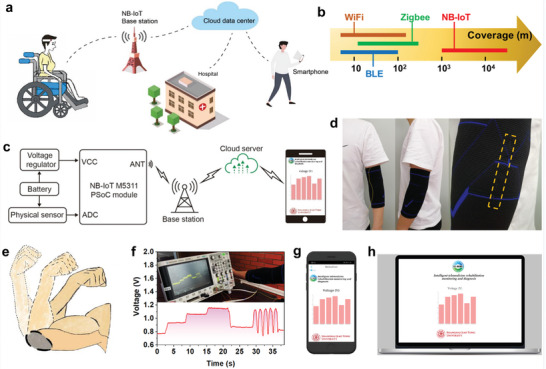
5G NB‐IoT system for intelligent telemedicine rehabilitation and diagnosis monitoring. a), Conceptual diagram of the intelligent telemedicine rehabilitation and diagnosis monitoring system. b), Communication distance comparison of the NB‐IoT with other communication technologies. c), System architecture of the 5G NB‐IoT system. d), The CSFS‐based fabric worn on the elbow. e), Bending deformation degrees of elbow indicating the movement of the rehabilitation condition of related diseases. f), Real‐time monitoring of elbow bending motion. g,h), Mobile terminals of mobile phones and computers for receiving and visually presenting the relevant health data.

Currently, faced with a shortage of public medical resources, telemedicine programs aim to transcend time and space constraints by establishing new connections between medical experts and patients. These programs enable telemedicine diagnosis as well as guided rehabilitation treatment and training, remotely accessible either at the hospital or at home. A suitable data acquisition and transmission method is key to achieving this goal. As shown in **Figure** **5**a, among the various remote communication technologies, 5G NB‐IoT stands out with its impressive communication distance of over 10 km. In contrast, communication systems based on ZigBee (<300 m),^[^ ^45^ ^]^ Wi‐Fi (<100 m),^[^ ^2^, ^46^ ^]^ and Bluetooth (1–20 m)^[^ ^47^ ^]^ have significant limitations for long‐distance transmission (Figure 5b). The proposed system is composed of a wearable CSFS, a communication module, an NB‐IoT base station, a cloud data center, and a mobile terminal. First, the CSFS worn on the human body collects different characteristic electrical signals under different rehabilitation actions; it can transmit sensor data from the sensors to the cloud server via the NB‐IoT network provided by the mobile operator, and the hospital or any personal mobile terminal can receive the health data instantly. Figure 5c depicts the system architecture of the 5G NB‐IoT system. First, the NB‐IoT programmed system‐on‐a‐chip (PSoC) module is registered with the NB‐IoT base station network through a radio frequency antenna (Figure S13, Supporting Information). After receiving the health information acquired by the CSFS, the PSoC payloads it into a message and uploads it to a cloud platform. Finally, the application software interface can be explored through the Android platform of the mobile terminal to access the cloud data center, which allows doctors to analyze and provide medical guidance over thousands of miles, thereby realizing intelligent telemedicine rehabilitation and diagnosis monitoring.

As a proof of concept, the CSFS was worn on the elbow to detect elbow joint movement (Figure 5d), and the degree of elbow flexion was used to illustrate its freedom and imitatively represent the rehabilitation conditions of related diseases (Figure 5e). During the application test, whether in static holding or dynamic reciprocating bending, the varying degrees of bending of the user's elbow were accurately recorded by the oscilloscope, demonstrating the excellent static and dynamic cooperative response ability of the CSFS, which is consisted with what has been discussed above (Figure 5f; Figure S14, Supporting Information). The relevant videos are presented in Videos S3 and S4 (Supporting Information) clearly. Moreover, Android APPs were designed on two mobile terminals of mobile phones and computers in this work for receiving and visually presenting the relevant health data (Figure 5g.h). As shown in Video S5 (Supporting Information), from data acquisition, and signal transmission to signal reception and reproduction, the entire system was operating normally, indicating that this system based on the CSFS and 5G NB‐IoT has great application potential in the field of intelligent telemedicine rehabilitation and diagnosis monitoring.

## 3 Conclusion

We developed a bionic flexible CSFS with high sensitivity and a large sensing range. The electromechanical changes in different stretching stages were simulated using COMSOL to explain the response mechanism of the sensor. The weavable CSFS is ideal for integration into wearable electronics for HMI and health monitoring, and their multifunctional capabilities were confirmed through our sensing and robotic control platforms. We further integrated the CSFS into an existing 5G NB‐IoT system and realized remote medical data collection, transmission, and reproduction, demonstrating its huge application prospects in the emerging field of high‐efficiency remote medical diagnosis. We hope that this work can prove valuable in the development of flexible electronics, especially in human–machine communication interfaces, leading to new opportunities for intelligent telemedicine rehabilitation and diagnosis monitoring in the context of an aging society and increasingly insufficient public medical resources.

## 4 Experimental Section

### Fabrication of Crack‐Spring Coupled Structure Fiber Sensor

The preparation of the CSFS followed the typical two steps. The spring‐shaped core–sheath fiber (Zhiheng Co., Ltd.) was combined by Polyethylene terephthalate (PET) fibers tightly wounding onto a highly elastic rubber fiber, serving as the highly stretchable substrate. Two‐scale conductive percolation networks were provided by multilayer graphene and silver paste respectively. First, the ultrasonic‐cleaned pure fiber (in deionized water for 1 h) were transferred into the multi‐layer graphene (Mgra) ink (1% weight concentration, Suzhou Tanfeng Graphene Technology Co., Ltd.), followed by ultrasonic treatment for 30 min. After drying in the oven (20 min at 80 °C), the first graphene conductive layer was constructed on outer PET spring fibers, creating a sensing foundation with kiloohm‐level conductivity for large‐range deformation monitoring. Second, the conductive silver paste (SPI 05001‐AB) was printed on the Mgra/fiber half side along the axial direction, and the resistance of Ag/Mgra/fiber drops directly to the several Ohms level. Due to the stress mismatch between the silver layer and the Mgra/fiber base layer, the crack will occur on the silver layer of the CSFS, generating an ultrahigh sensitivity of the sensor.

### Electro‐Mechanical Characterization

The resistance of the CSFS was recorded in situ using LCR meter analyzer (WAYNE KERR No. 4100). The voltage signals were acquired by the digital oscilloscope (DSO‐X 2024A, Agilent). The dynamic mechanics tests were performed using an electronic universal tensile testing machine (AGS‐X, SHIMADZU), which can be set to different motion modes for both stretching and relaxing stages, such as strain rate, strain amplitude, *etc*. Generally, the variation of relative resistance (*ΔR/R_0_*) was adopted to define the electromechanical characteristics of the CSFS. Specifically, *ΔR* = *R* − *R_0_*, in which *R*_0_ and *R* were the resistances of the CSFS before and after deformation, respectively. The sensitivity was described by gauge factor (GF), which is calculated by the equation GF = *ΔR/R_0_/*strain.

### Design of Human‐Machine System of Gesture Control

The fabricated CSFS sewn into the outer side of the finger knuckles of the glove can perceive finger movements to generate a quantitative pulse electrical signal, which can be used to build a HMI platform through the peripheral circuit modules. First, the quantitative analog signals converted by finger motions were fed into an analogto‐digital converter (ADC). If the voltage amplitude was higher than a specific threshold, another micro‐controller will process the signal and send corresponding instructions to the executive module, which will control the robotic hand to make the same movements as a human finger. Based on these design circuits, the system could realize gesture recognition and control.

### Design of the 5G NB‐IoT System

The NB‐IoT PSoC module (M5311, Longmain Co., Ltd.) was programmed in the Microsoft Visual Studio IDE, which could collect the voltage signal generated by the CSFS sewn at the elbow part through ADC. Then, the required data could be payloaded as a message by the PSoC and uploaded into the cloud platform with the Lightweight M2M (LWM2M) protocol. The detailed information can be obtained in previous research work.^[^ ^48^ ^]^

### Characterizations and Measurements

The microscopic morphologies were carried out by field emission scanning electron microscopy (FE‐SEM, ZEISS Gemini), field emission transmission electron microscopy (TEM, TALOS F200X), and atomic force microscopy (AFM, Nanonavi E‐Sweep), respectively. The structural characterizations of Mgra and the CSFS were performed by Raman spectroscopy (TESCAN‐MAIA3), and X‐ray diffraction (XRD, D8 ADVANCE Da Vinci), respectively. The ultrasonic treatment during the cleaning process and the graphene modification process was conducted by an ultrasonic cell disruptor (JY88‐II), and the ultrasonic process works for 3 s and stops for 2 s alternately.

## Conflict of Interest

The authors declare no conflict of interests.

## Author Contributions

B.Y. and L.J.L. conceived and designed the experiments. L.J.L., and G.S.H. carried out experiments and collected the overall data. L.J.L. contributed to materials fabrication and characterization. L.J.L. performed simulation, electrical properties characterization and worked on sensors demonstration. G.S.H. designed and analyzed the circuit part. L.J.L. and B.Y. analyzed all the data and wrote the paper. All authors discussed the results and commented on the manuscript.

## Supporting information

Supporting Information

Supplemental Video 1

Supplemental Video 2

Supplemental Video 3

Supplemental Video 4

Supplemental Video 5

## Data Availability

The data that support the findings of this study are available from the corresponding author upon reasonable request.
