# Supplementary material for: 5G NB‐IoT System Integrated with High‐Performance Fiber Sensor Inspired by Cirrus and Spider Structures
Source: Adv Sci (Weinh). 2024 Mar 9;11(18):2309894. doi: 10.1002/advs.202309894 (PMC11095228; doi:10.1002/advs.202309894)
Supplement: Supplementary file 1 — Supporting Information [file ADVS-11-2309894-s005.pdf]

ADVANCED SCIENCE
Open Access

Supporting Information

for *Adv. Sci.*, DOI 10.1002/advs.202309894

5G NB-IoT System Integrated with High-Performance Fiber Sensor Inspired by Cirrus and Spider Structures

*Lijun Lu, Guosheng Hu, Jingquan Liu and Bin Yang**

Supplementary information for

5G NB-IoT System Integrated with High-Performance Fiber Sensor Inspired by Cirrus and Spider Structures

Lijun Lu[1,2,3], Guosheng Hu[2,3], Jingquan Liu[2] and Bin Yang[2]*

[1]Key Laboratory of Materials Physics of Ministry of Education, School of Physics and Microelectronics, Zhengzhou University, Zhengzhou 450001, China

[2]National Key Laboratory of Science and Technology on Micro/Nano Fabrication, Shanghai Jiao Tong University, Shanghai, 200240, China

[3]Department of Micro/Nano Electronics, School of Electronic Information and Electrical Engineering, Shanghai Jiao Tong University, Shanghai, 200240, China

*Corresponding author: binyang@sjtu.edu.cn

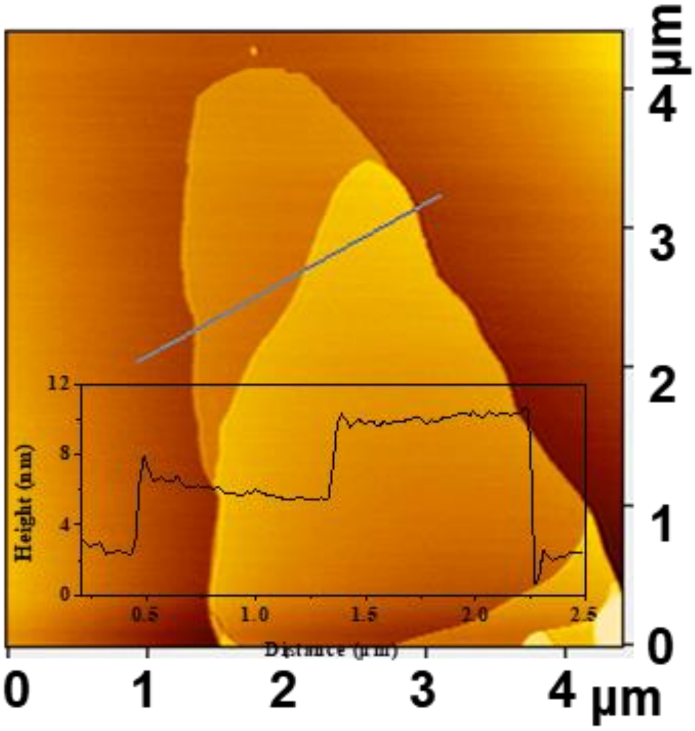

Figure S1. AFM image of the Mgra.

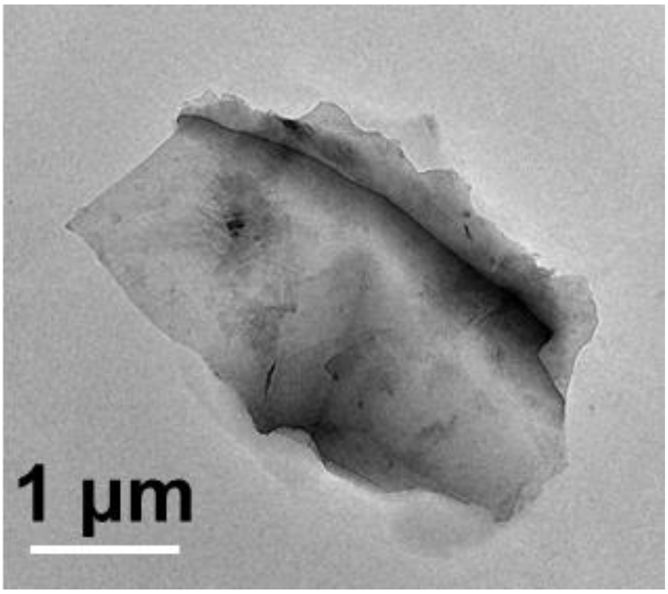

Figure S2. TEM image of the Mgra.

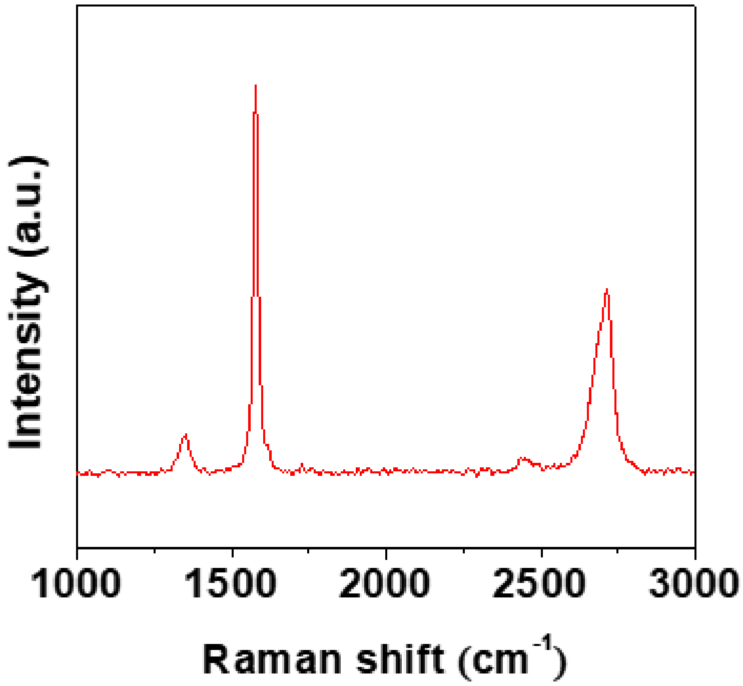

Figure S3. Raman spectra of the Mgra.

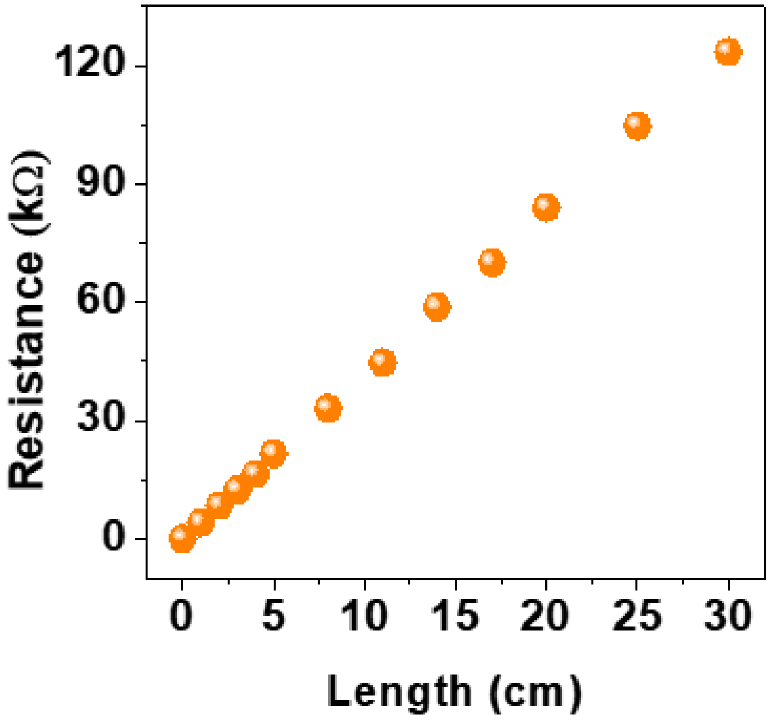

Figure S4. The relationship between resistance and test length of Mgra/fiber.

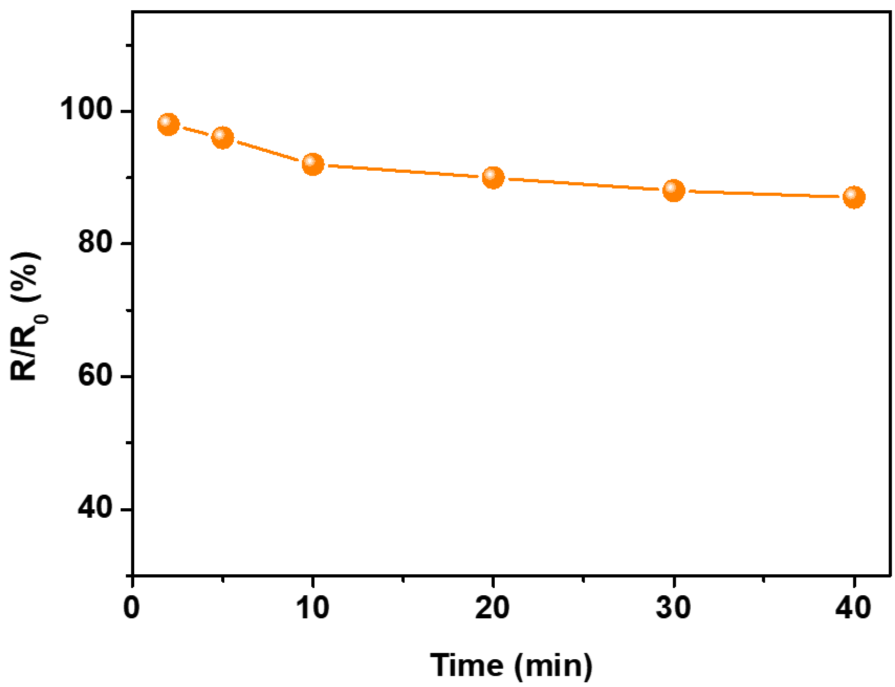

Figure S5. Relative resistance change of the Mgra/fiber with the treatment time of washing machine.

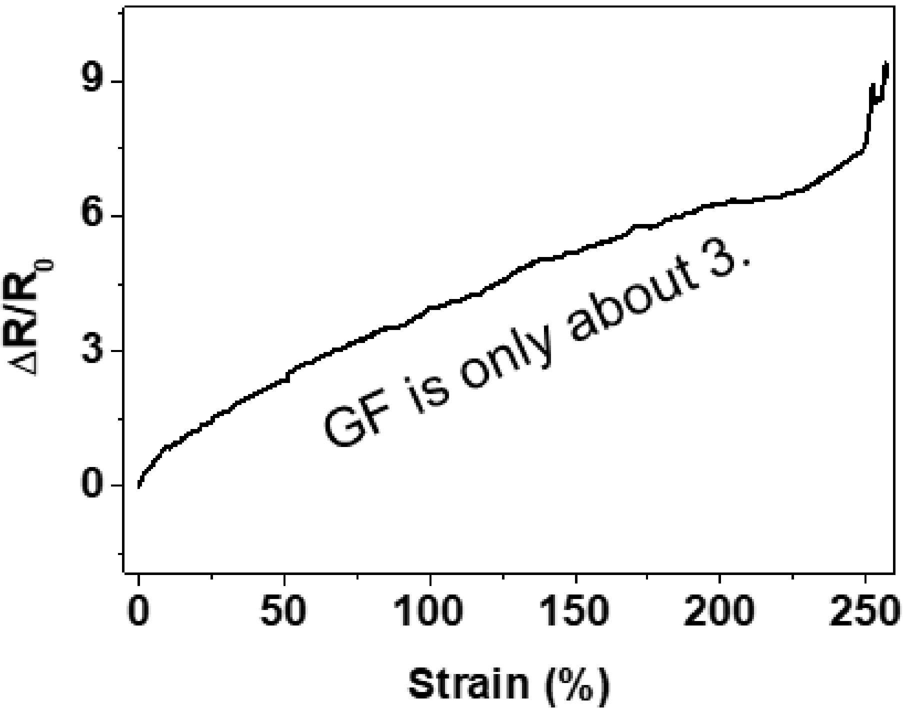

Figure S6. Typical relative resistance-strain curve of the Mgra/fiber.

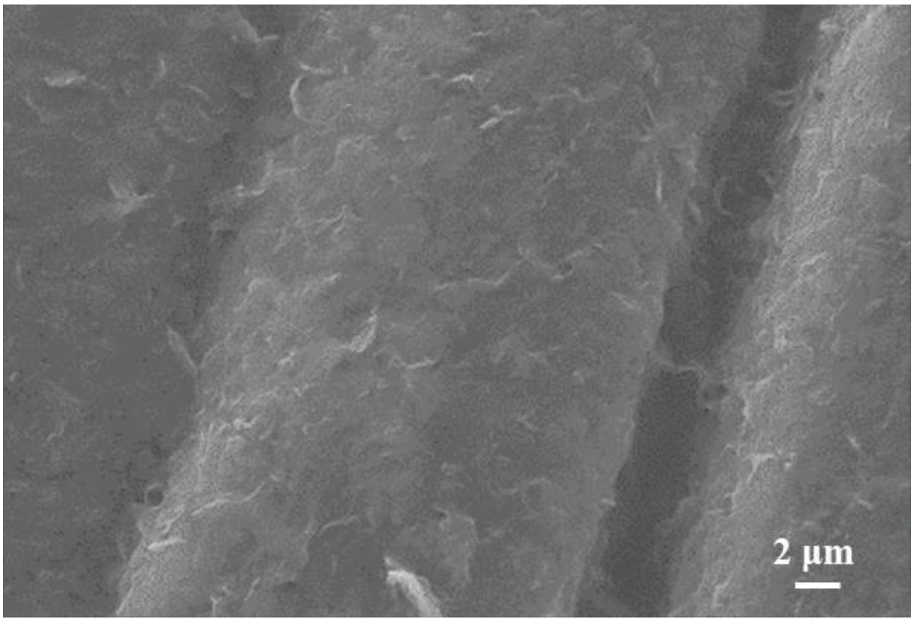

Figure S7. The SEM image of the Mgra/fiber.

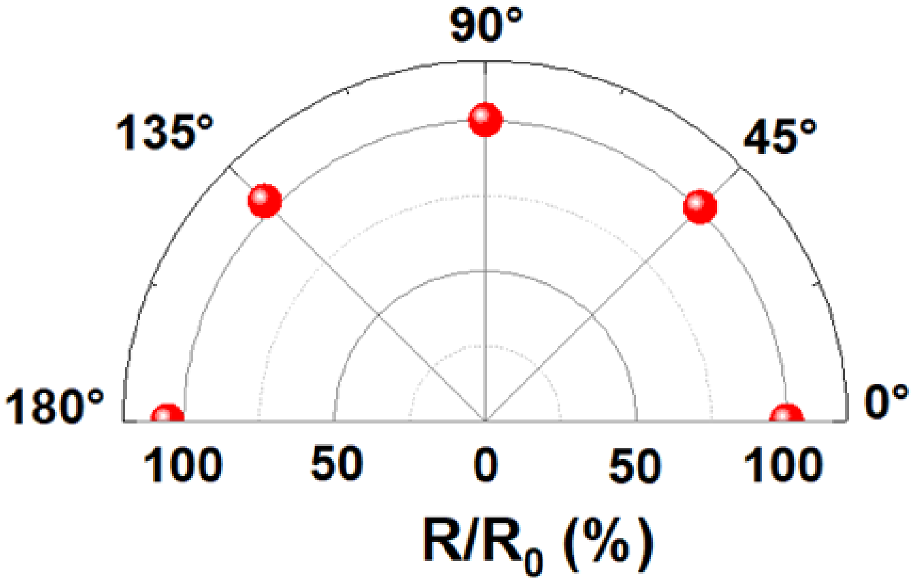

Figure S8. The effect of bending angle on the resistance of the Mgra/fiber.

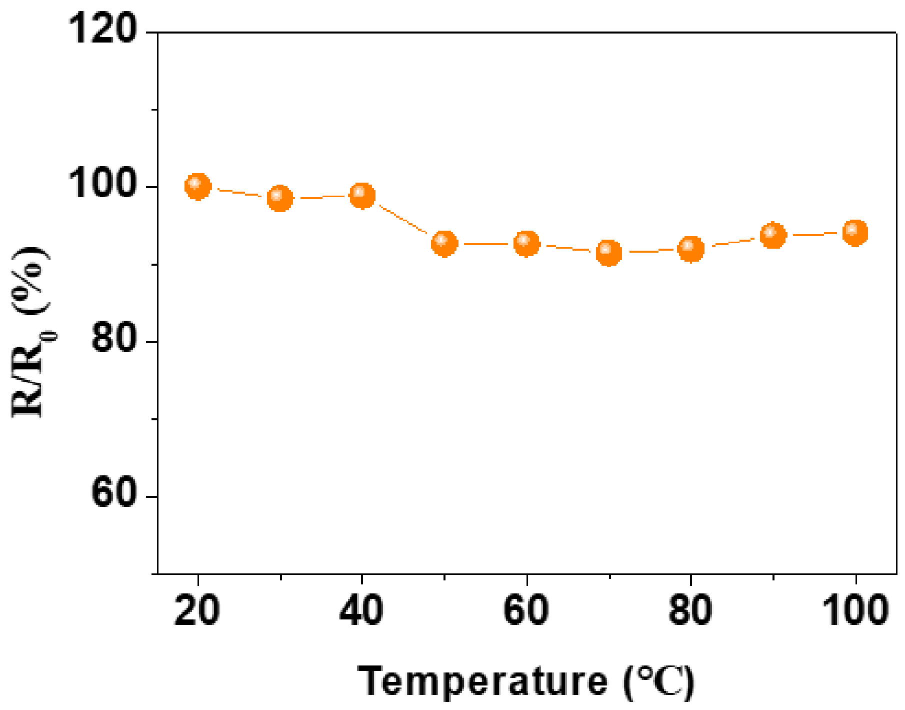

Figure S9. Tolerance to temperature of the CSFS.

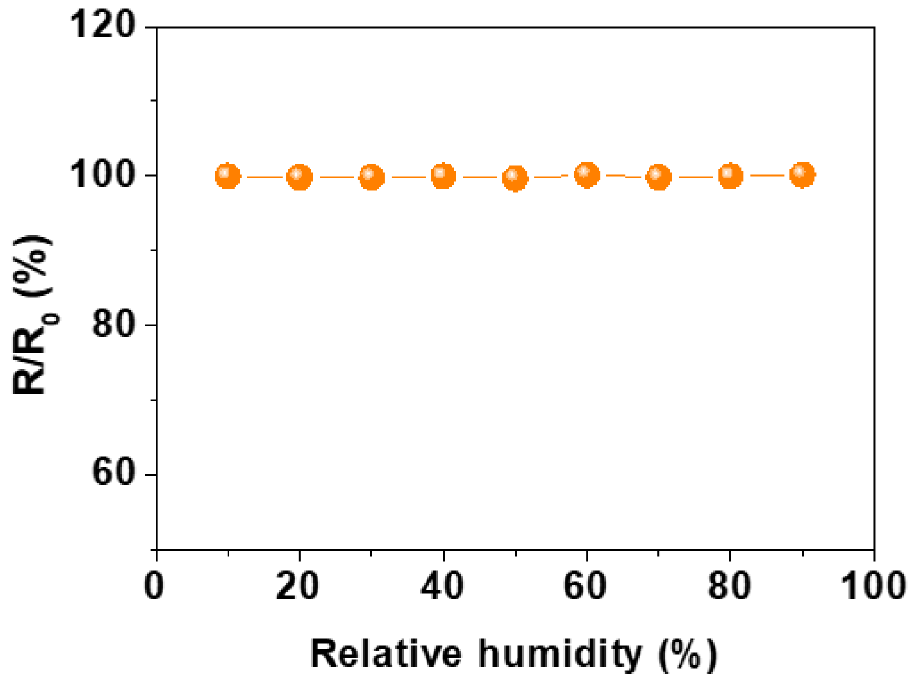

Figure S10. Tolerance to Relative humidity of the CSFS.

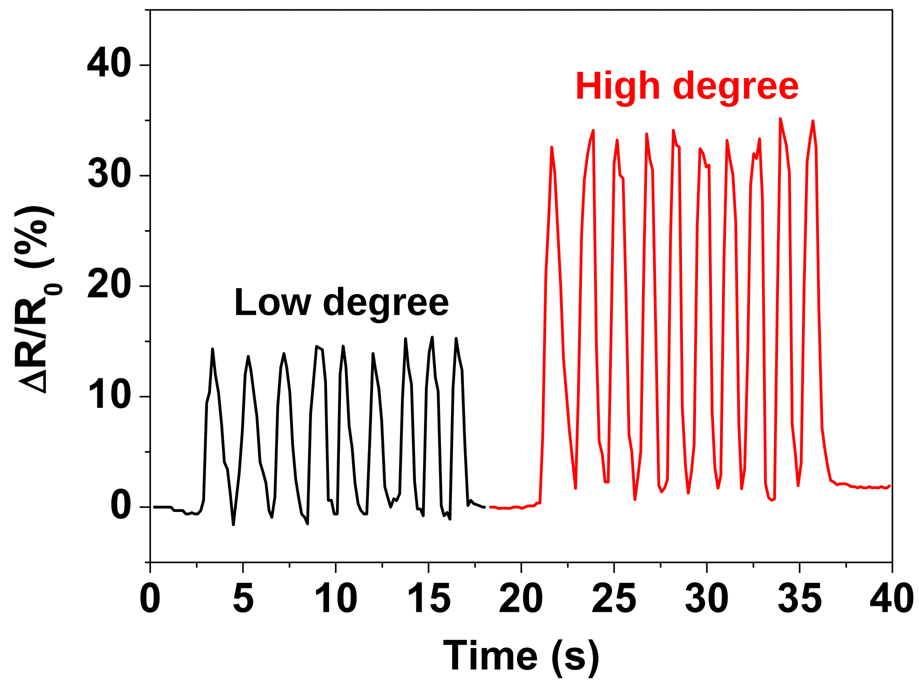

Figure S11. Real-time monitoring of finger bending motion in simulate hand sweating state.

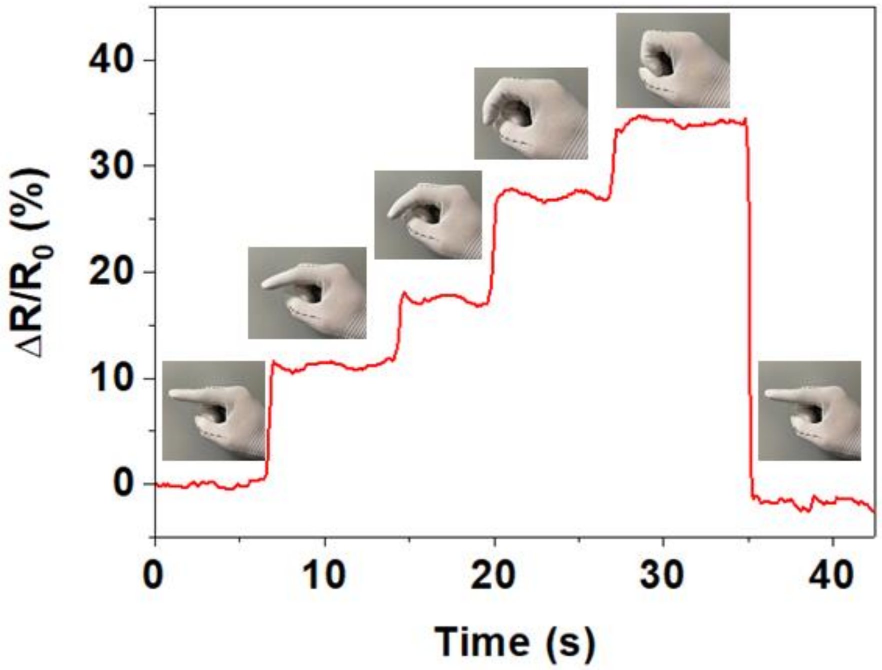

Figure S12. Real-time monitoring of finger bending motion.

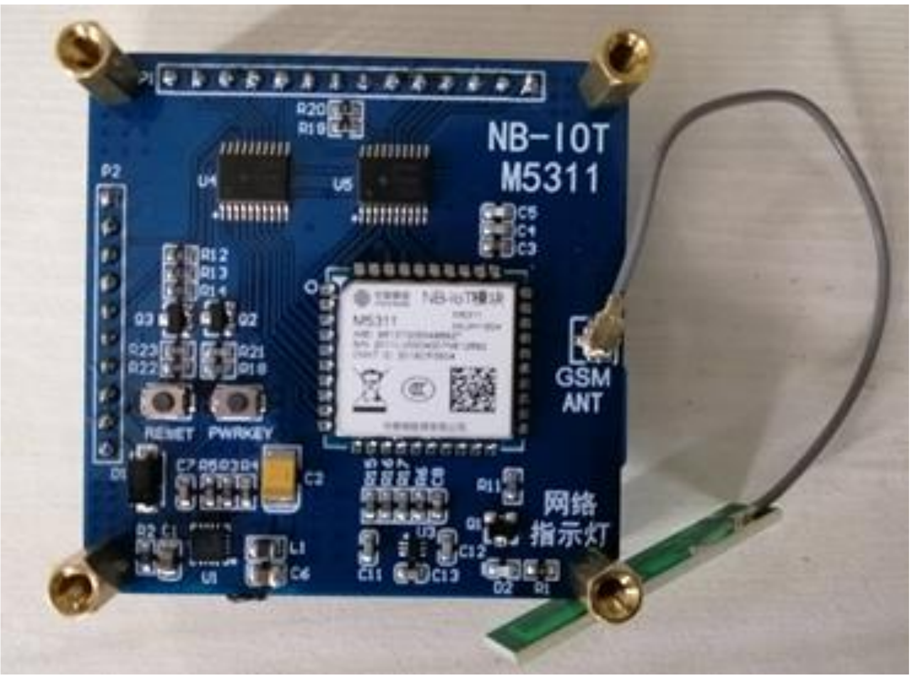

Figure S13. NB-IoT system M5311 Development Board.

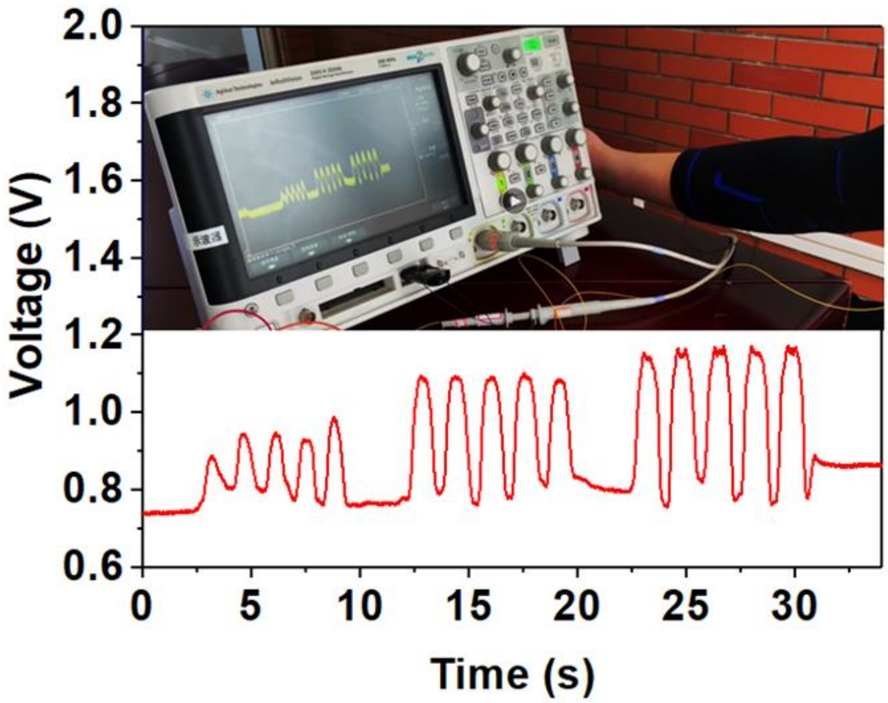

Figure S14. Real-time monitoring of elbow bending motion.
